# Integrating X-ray phase-contrast imaging and histology for comparative evaluation of breast tissue malignancies in virtual histology analysis

**DOI:** 10.1038/s41598-024-56341-6

**Published:** 2024-03-09

**Authors:** Sandro Donato, Lucia Mariel Arana Peña, Fulvia Arfelli, Luca Brombal, Luisella Colmo, Renata Longo, Fulvia Martellani, Giuliana Tromba, Fabrizio Zanconati, Deborah Bonazza

**Affiliations:** 1 Department of Physics, University of Calabria, 87036 Rende, CS Italy; 2 Division of Frascati, INFN, 00044 Frascati, RM Italy; 3 Department of Physics, University of Trieste, 34127 Trieste, Italy; 4 Division of Trieste, INFN, 34127 Trieste, Italy; 5 Elettra-Sincrotrone Trieste S.C.p.A, 34149 Trieste, Italy; 6 Unit of Surgical Pathology of the Cattinara Hospital, Azienda Sanitaria Universitaria Giuliana Isontina (ASUGI), 34149 Trieste, Italy

**Keywords:** Imaging techniques, Breast cancer, Computed tomography, Imaging techniques, Breast cancer, Computed tomography

## Abstract

Detecting breast tissue alterations is essential for cancer diagnosis. However, inherent bidimensionality limits histological procedures’ effectiveness in identifying these changes. Our study applies a 3D virtual histology method based on X-ray phase-contrast microtomography (PhC $\mu$CT), performed at a synchrotron facility, to investigate breast tissue samples including different types of lesions, namely intraductal papilloma, micropapillary intracystic carcinoma, and invasive lobular carcinoma. One-to-one comparisons of X-ray and histological images explore the clinical potential of 3D X-ray virtual histology. Results show that PhC $\mu$CT technique provides high spatial resolution and soft tissue sensitivity, while being non-destructive, not requiring a dedicated sample processing and being compatible with conventional histology. PhC $\mu$CT can enhance the visualization of morphological characteristics such as stromal tissue, fibrovascular core, terminal duct lobular unit, stromal/epithelium interface, basement membrane, and adipocytes. Despite not reaching the (sub) cellular level, the three-dimensionality of PhC $\mu$CT images allows to depict in-depth alterations of the breast tissues, potentially revealing pathologically relevant details missed by a single histological section. Compared to serial sectioning, PhC $\mu$CT allows the virtual investigation of the sample volume along any orientation, possibly guiding the pathologist in the choice of the most suitable cutting plane. Overall, PhC $\mu$CT virtual histology holds great promise as a tool adding to conventional histology for improving efficiency, accessibility, and diagnostic accuracy of pathological evaluation.

## Introduction

Breast cancer is one of the most common cancers worldwide, particularly among women. While the incidence rates of cancer can vary depending on factors such as geography, age, and population, breast cancer generally ranks among the top in terms of occurrence, with an estimated 2.3 million new cases per year, representing 11.7$\%$ of all cancer cases^1,2^. According to global cancer statistics, breast cancer is the most frequently diagnosed cancer in women and it is the fifth leading cause of cancer mortality worldwide^2^. Among women, breast cancer accounts for 1 in 4 cancer cases and for 1 in 6 cancer deaths, ranking first for incidence and mortality in the vast majority of countries. According to the recommendations of the American Cancer Society^3^ different tests can be used to diagnose breast cancer, such as mammography, breast ultrasound, breast magnetic resonance imaging, digital breast tomosynthesis, and dedicated breast computed tomography (bCT)^4,5^, each modality playing an important role and having its advantages and limitations, the main problem being the lack of specificity to discern among the different types of invasive breast cancers^6^.

Based on previously mentioned radiological images and clinical exams, patients suspected of having breast cancer are referred to undergo a breast surgical biopsy and, eventually, molecular testing. In confirmed cancer cases, treatment options include breast-conserving surgery (lumpectomy) or complete breast removal (mastectomy) to eliminate cancerous or abnormal breast tissues. These tissues are then examined by pathologists. Lumpectomy is considered safe^7^ and the preferred choice for early-stage cancers since it offers comparable survival rates to mastectomy while providing better cosmetic and psychological outcomes. During this procedure, a small amount of normal tissue surrounding the tumor, known as the surgical margin, is also removed and examined through histology to determine if cancer is present between the tumor and the outer edge of the margin. The decision between mastectomy and lumpectomy is influenced by several factors, including the size and location of the tumor, the stage of cancer, the individual’s overall health, and personal preferences. The choice is often made through a collaborative discussion between the patient and their healthcare team.

The breast has three main components: the skin, subcutaneous tissue, and breast tissue. The breast tissue consists of adipose, connective, and glandular tissue, the latter also known as the parenchyma. It is a complex network of epithelial and stromal components, with lobes and milk ducts embedded within it. The stroma, which makes up the largest portion of breast volume in non-lactating adults, contains varying proportions of fibrous and adipose tissue depending on age and individual differences^8^. The glandular epithelium comprises approximately 10–15$\%$^9^ of the adult female breast and is composed of 15–20 lobes, which are subsequently composed of several lobules. These lobules, known as terminal ductal lobular units (TDLU), are clusters of milk-producing glands that serve as the functional and structural units of the breast. The glandular tissue and ductal-lobular systems of the breast play a crucial role in breast health assessments and the diagnosis of breast cancer, as different breast lesions typically originate from these areas. In this study, normal breast tissue and three lesions originating from the ductal-lobular system were investigated: intraductal papilloma, invasive lobular carcinoma, and micropapillary intracystic carcinoma. Each lesion exhibits distinct presentation, morphology, and clinical behavior. Intraductal papillomas are benign breast neoplasms that develop within mammary ducts. They are composed of breast epithelium, supported by underlying stroma and a branching fibrovascular core. The latter plays a crucial role in providing support, nourishment, and function to the breast tissue. Changes in the appearance or structure of the fibrovascular core can be indicative of breast health issues. Intraductal papillomas are relatively rare, with an incidence of 2–3$\%$ of all breast carcinomas, and are commonly diagnosed in women aged 30–55 years. On the other hand, papillary lesions are observed in up to 5$\%$ of breast core needle biopsies^9^. Micropapillary intracystic carcinoma is a rare subtype of breast cancer characterized by cancer cells forming finger-like projections within cyst-like spaces^10,11^. It tends to be more aggressive compared to other breast cancers, potentially spreading to lymph nodes and other parts of the body. The invasive (or infiltrating) lobular carcinoma (ILC) is the second most common breast carcinoma^12^. ILC is characterized by non-cohesive cells infiltrating adjacent healthy breast tissues in a single-file pattern, often without forming a palpable lesion. This makes detection challenging during physical exams^6^, and current imaging techniques have limited specificity in distinguishing ILC from other invasive breast cancers^13–15^. The architecture of the ducts is typically preserved in invasive lobular carcinoma cells, which reduces the sensitivity of mammography, leading to higher false-negative rates during radiological diagnosis^6^.

Histology is the gold standard for the evaluation and characterization of breast tissues. Owing to the great variety of class-specific dyes (DNA, proteins, lipids, or carbohydrates), histology provides high discriminative power at both tissue and cellular levels^16^. Microscopy, with its high spatial resolution (better than 1 μm), enables imaging at the level of individual cells. However, histological assessment involves slicing, staining, imaging, and analysis, being a challenging, time-consuming, and costly process^17^. Additionally, histology is inherently a two-dimensional technique, even though serial sectioning can offer some depth information^18^. Nonetheless, the slicing procedure may introduce tissue deformation or cutting artifacts^19,20^. To address these limitations, the concept of virtual histology has emerged, offering non-destructive, three-dimensional imaging with sufficient soft tissue sensitivity and spatial resolution. X-ray micro-computed tomography ($\mu$CT) presents itself as a valuable option, thanks to its ability to capture three-dimensional images at a micrometer scale using X-rays. However, conventional $\mu$CT struggles to differentiate soft tissues due to their similar X-ray attenuation properties. Contrast-enhanced $\mu$CT has been proposed as a solution, where tissue-specific radio-opaque contrast agents are perfused into histological samples to enhance image contrast^21^. Alternatively, phase-contrast (PhC) $\mu$CT has gained attention as a promising technique that does not require a dedicated tissue staining, making it simpler and compatible with standard histology procedures. X-ray PhC $\mu$CT combines the soft tissue sensitivity of X-ray PhC imaging with tomographic reconstruction to visualize and analyze the internal structure of biological tissues at a microscopic level in 3D. It has been demonstrated to provide detailed information on tissue morphology, cellular structures, and the distribution of components within the sample^19,22–28^. Advanced X-ray PhC $\mu$CT systems and techniques have achieved sub-micrometer resolution, enabling the visualization of fine structures within biological samples^29,30^. Among the different phase-contrast techniques available, the propagation-based technique^31^ is experimentally straightforward, requiring to position sample and detector at a distance in the order of tens of centimeters for high-resolution scans^28^. However, the propagation-based technique demands strict X-ray source coherence, making synchrotron radiation facilities ideal for this type of imaging^32–37^.

Within this context, the objective of this study is to demonstrate the potential of PhC $\mu$CT-based virtual histology in highlighting specific and morphologically relevant features of three distinct surgical samples with different patterns of presentation, morphology, and clinical behavior. By visualizing tissue microstructure, researchers and clinicians can gain valuable insights into cellular interactions, tissue architecture, and disease processes, contributing to a deeper understanding of biological systems. To facilitate direct comparison, an imaging protocol enabling experienced pathologists to examine histology slices at varying spatial resolutions is introduced. The visualization advantages and disadvantages of PhC, compared to histology using different staining protocols, are discussed in relation to specific features such as the stromal/epithelium interface, microcalcifications, identification of individual cellular elements, and the fibrovascular core. Lastly, this study concludes with the presentation of high-resolution 3D renderings showcasing the intricate volume morphology of the tissue samples. The most representative images, highlighting the visibility of key features in each breast tissue, are presented. This preliminary investigation represents the initial phase of a pioneering approach towards a systematic virtual histology study including a larger number of lesion types. The ultimate goal is to proof the added value PhC $\mu$CT-based virtual histology in evaluating the invasiveness of malignant diseases, not only in breast tissues, thus prompting its translation toward clinics.

## Results

### Normal breast tissue

**Figure 1:**
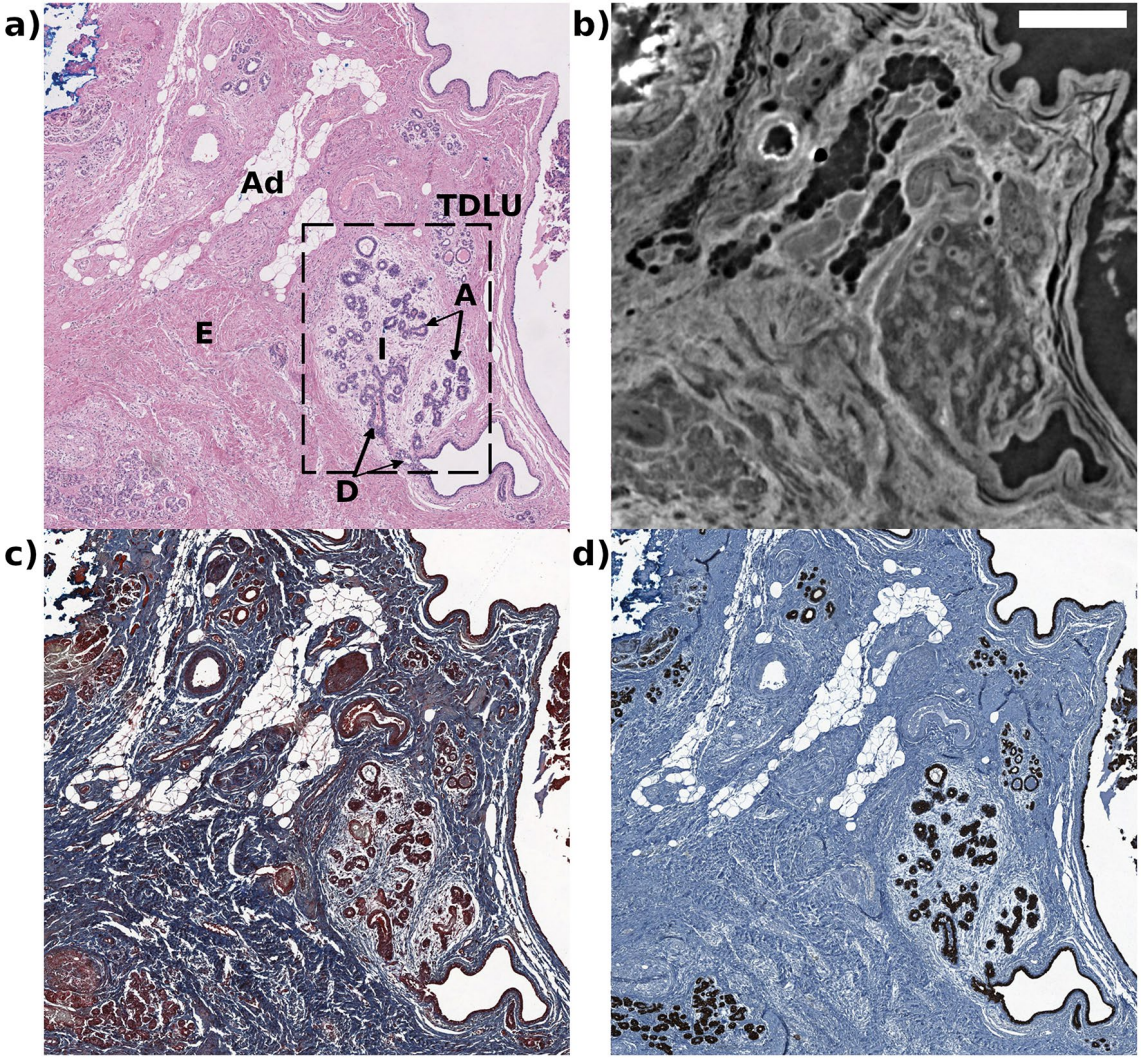
A comprehensive visual correlation of the mammary lobules (the functional units of the mammary gland) and fibroadipose tissue between the histological staining techniques (**a**,**c**,**d**), obtained with a D-Sight F 2.0 slide scanner for digital pathology, and PhC $\mu$CT (**b**) at 4 μm. The histological techniques presented are: (**a**) H&E, (**c**) Masson’s trichromic, and (**d**) pancytokeratin. In the panel (a) there are two lobes, each one consisting of approximately twenty small glandular structures called acini (A) that open into a terminal duct (D). There are also indicated: adipocytes (Ad), intralobular stroma (I), interlobular stroma (E), and the terminal duct lobular unit (TDLU) inside the dashed box. In the upper left corner of each image is also visible the cauterization artifact that appears blue in H&E staining and white in PhC $\mu$CT images. The scalebar equals to 0.5 mm.

Figure 1 shows a representative section of the reconstructed PhC $\mu$CT dataset of the breast biopsy specimen, taken from an uninvolved site from a patient diagnosed with intraductal papilloma, along with corresponding histology slides of the 2D haematoxylin and eosin (H&E) tissue (Fig. 1a), Masson’s trichrome (Fig. 1c) and pancytokeratin stainings (Fig. 1d). Observing the H&E image and the corresponding PhC $\mu$CT image, broad features such as cellular and stromal density, arrangement, size, and organization may be assessed. Based on these characteristics, mammary lobules can be identified in each image. The mammary lobules, which are observed at the ends of the ducts, consist each of approximately twenty small glandular structures called acini (A) that open into a terminal duct (D). The rectangle outlined with the dashed line in Fig. 1a depicts TDLU. Ducts and mammary lobules are surrounded by connective tissue, blood, and lymphatic vessels, nerves, adipose tissue (Ad) which provide nutrition and support^38^. The TDLUs are embedded in specialized, hormonally responsive connective tissue stroma called intralobular stroma (I), consisting primarily of fibroblasts. This intralobular stroma is subsequently surrounded by a more compact interlobular stroma (E). The intralobular stroma is usually looser and more cellular than the interlobular stroma, and unlike the latter, it usually does not have adipose tissue^39^. Intralobular stroma is hormone sensitive and shows cyclic histologic changes. In this sample, the intralobular stroma is edematous in response to hormonal changes, the difference in density is clearly visible in all four panels. Adipose tissue is abundant in the interlobular spaces, while its presence in the intralobular stroma is scarce. This tissue is recognized very well in H&E and PhC $\mu$CT histology, it is possible to identify the single adipocyte cells in both images. Differentiating the individual parts of normal tissue is very useful to subsequently identify both benign and malignant alterations. It is useful to identify the basement membrane and the interface zone between the epithelium and the stroma. The normal breast tissue sample was scanned at 4 μm and 2.5 μm. The architecture of the TDLU was observed as a highly defined organized structure, already at a pixel size of 4 μm as can be seen in Fig. 1. It was possible to distinguish configurations such as ducts and acini in a loose stromal context. Although PhC $\mu$CT images did not show single ductal cells as in the histological staining techniques, they did offer enhanced visibility of the ductal lobular unit compared to any of the three histological images, without requiring specific preparation. In particular, phase-contrast images improve the visibility of the TDLU margin that appears clearly outlined as opposed to the color gradient that seen in any of the histological colorations. The differences observed between a physiological intralobular and an interlobular stroma in conventional and virtual histology images are also visible. For normal tissue, the proposed method is comparable to histology and for some structures, it provides improved visibility.

### Intraductal papilloma

**Figure 2:**
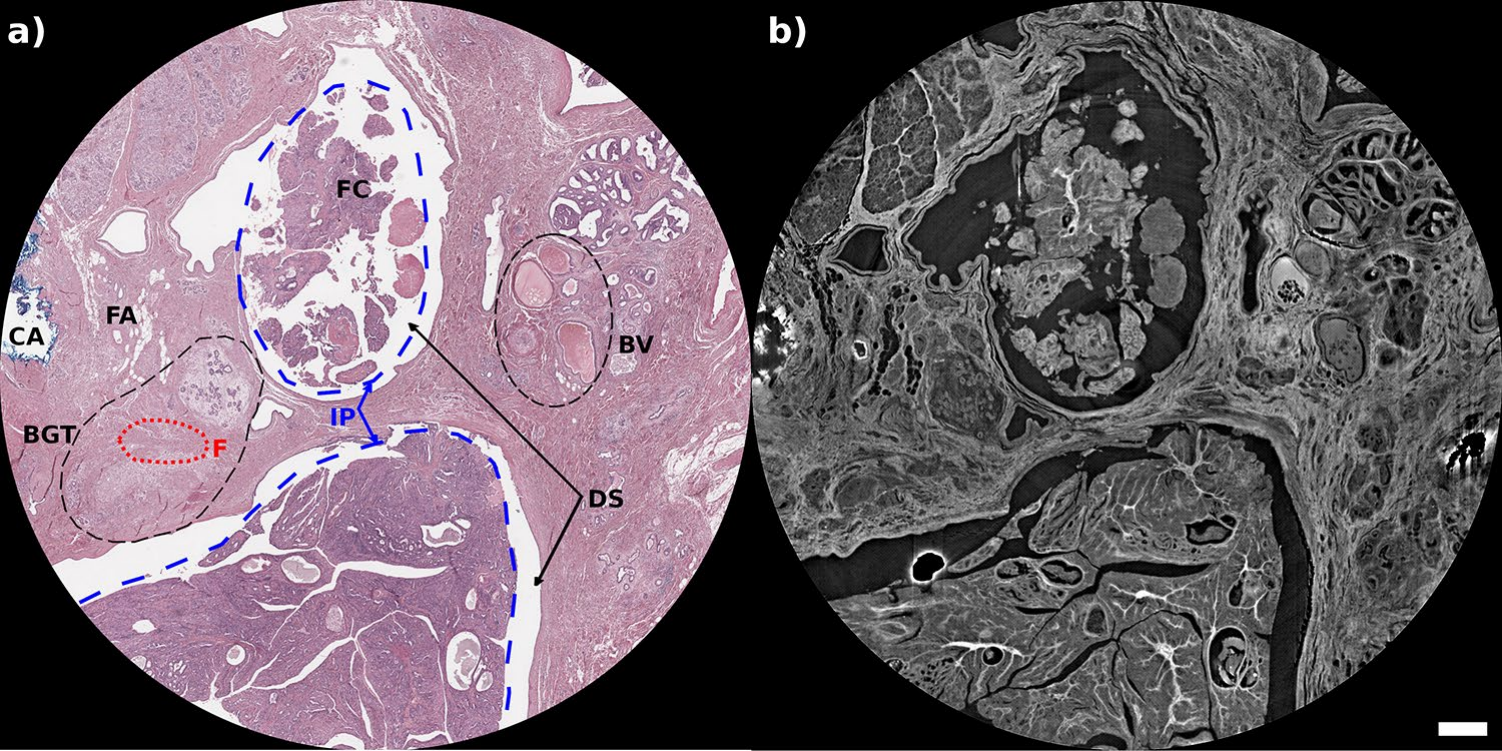
Region of histological features of interest for intraductal papilloma as seen in conventional hematoxylin and eosin staining (**a**) and PhC $\mu$CT (**b**) with a pixel size of 4 $\mu$m. In H&E image are also shown: intraductal papilloma portion (IP) inside the dashed blue lines, ductal space (DS), blood vessels (BV), the fibroadipose tissue (FA), elastic collagen and fibers (F) inside the red dotted line, the normal breast glandular tissue (BGT), cauterization artifacts (CA) due to electrosurgical cut of the tissue, and (FC) denotes the fibrovascular cores (FC). The scalebar is 0.5 mm.

Intraductal papilloma is a benign proliferative lesion of the breast. Histologically, complex arborizing fibrovascular cores lined by myoepithelial cells and covered by luminal cells are present within a dilated ductal space (DS in Fig. 2).

The side-by-side presentation of PhC $\mu$CT and histology data shown in Fig. 2 enables histology-guided identification of a range of tissue structures and diagnostically relevant histological criteria. In this example, key microstructural features, such as the normal breast glandular component (BGT), intraductal papilloma portions (IP, denoted as dashed blue lines), blood vessels (BV), and fibroadipose tissue (FA), can be clearly seen also in the $\mu$CT images when compared to histological sections. In PhC $\mu$CT images, the stromal component is better distinguished from different components. Indeed, in Fig. 2, but also in Fig. 1, it can be seen how dense interstitial components composed of collagen can be distinguished from less dense components such as elastic fibers (F), and the fibroblasts (red dotted line in Fig. 2). One area of cautery artifacts (CA) is also shown in Fig. 2. Looking at the upper-left corner in each panel of Fig. 1, different types of electrosurgical lesions can be found in these areas such as complete charring without cellular structures, severe tissue degeneration, confluent tissue with few discernible structures, indistinguishable nuclei, rupture tissue, distressed cellular architecture with a thin appearance, irregular elongated and tapering nuclei, smeared chromatin, visible distorted fibroblast nuclei without clear cell boundaries, and cells visible but not clear whether they are epithelial or stromal. These artifacts are visible in PhC $\mu$CT as intense and irregular white bands.

### Micropapillary intracystic carcinoma

**Figure 3:**
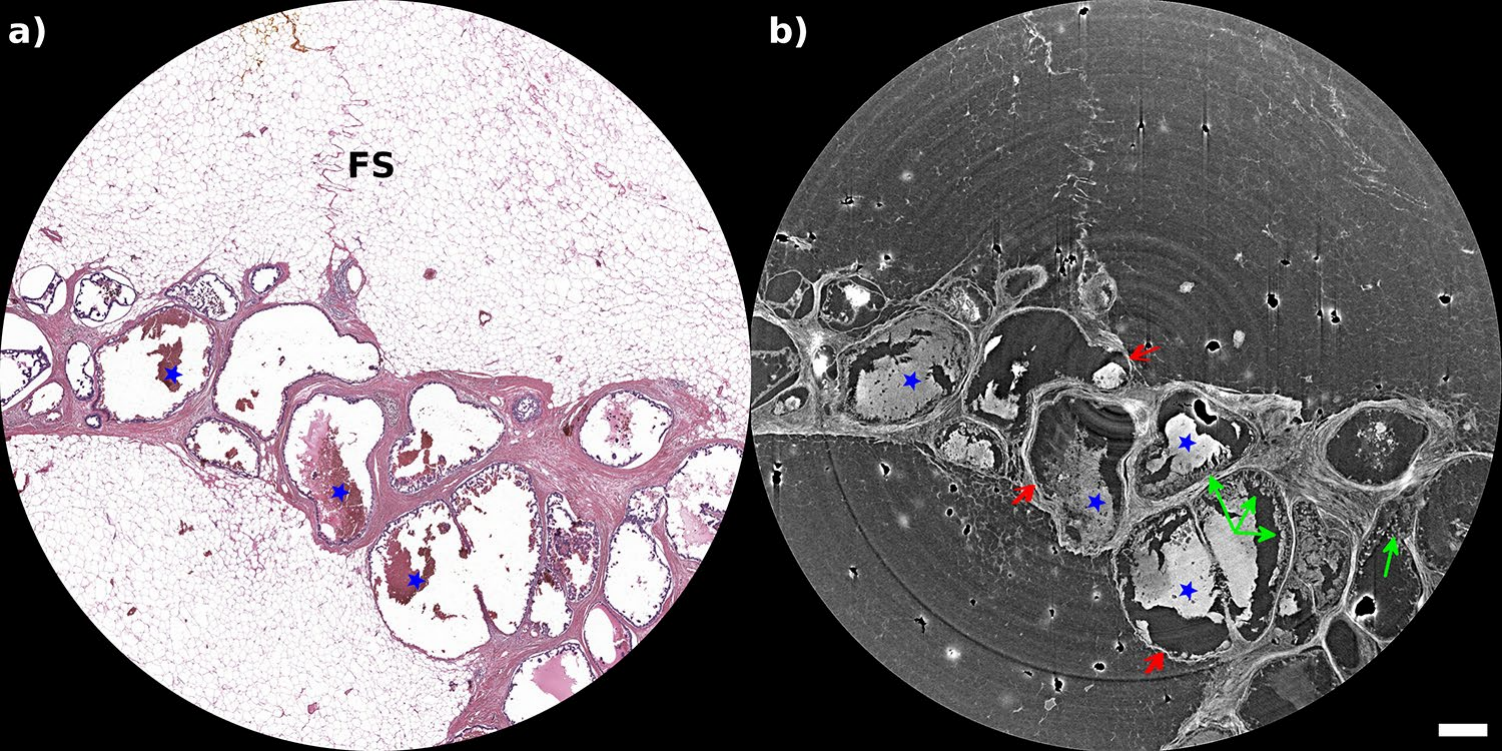
Region of histological features of interest for invasive micropapillary cystic carcinoma as seen in conventional haematoxylin and eosin staining (**a**) and PhC $\mu$CT (**b**) with a pixel size of 4 μm. The images show the fibrous septum (FS), neoplastic micropapillae of ductal epithelium (green arrows), accumulation of packed red blood cells (blue stars), and basement membrane (red arrows), the latter being an important indicator of breast cancer progression. The scalebar is 0.5 mm.

Virtual histology images of the micropapillary cystic carcinoma show an enhancement between the stromal/epithelial interface and the basement membrane (red arrows in Fig. 3), which is a key indicator of breast cancer progression. In addition, neoplastic micropapillae of ductal epithelium (indicated by green arrows) and accumulations of packed red blood cells (blue stars) are clearly visible.

By visualizing the micropapillary cystic carcinoma in three dimensions, virtual histology allows for a comprehensive examination of the tumor’s spatial distribution, extent of invasion, and interactions with surrounding tissues. The enhancement observed between the stromal/epithelial interface and the basement membrane can be precisely localized and quantified, providing a more accurate assessment of tumor progression and invasiveness, aiding in personalized treatment decisions, and improving patient outcomes. Even very small and thin structures such as the fibrous septum (FS), were clearly identified in the PhC $\mu$CT images at a pixel size of 4 μm. Clots within the cystic spaces are clear in PhC $\mu$CT, while they may not be present in the H&E images, because these structures can be lost during the specimen cutting procedure or during glass slice preparation. For this breast lesion, PhC virtual histology is overall comparable to histology while the interface is better depicted than in conventional H&E images.

### Invasive lobular carcinoma

The invasive lobular carcinoma of the breast (ILC) is the second most frequent histological type, accounting for 10–15$\%$ of all invasive breast carcinomas. Radiological diagnosis of ILC can be particularly challenging, and higher false-negative rates (up to 19$\%$) are reported for ILC than for other invasive cancers at mammography^6^.

**Figure 4:**
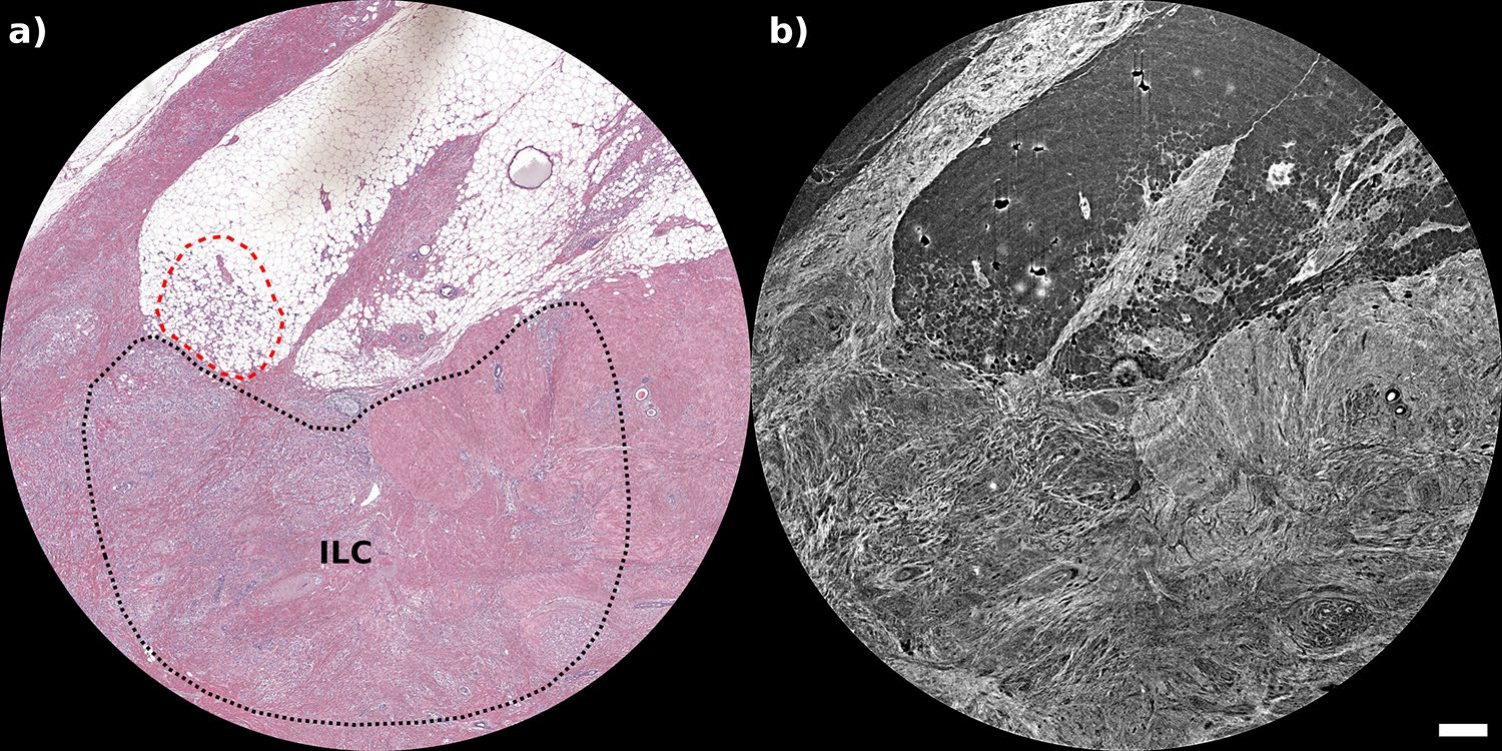
Region of histological features of interest for invasive lobular carcinoma, as seen in conventional H&E staining (**a**) and PhC $\mu$CT (**b**) with a pixel size of 4 μm. The adipose component as well as the stromal component infiltrated by lobular carcinoma, respectively, included in red and black dashed lines, are well distinguishable in both images. The scalebar is to 0.5 mm. The white spots in CT reconstructions (in correspondence of the area marked in red in panel **a**) are attributable to air bubbles in the paraffin at different vertical planes, primarily resulting from the sharp interface between paraffin and air.

**Figure 5:**
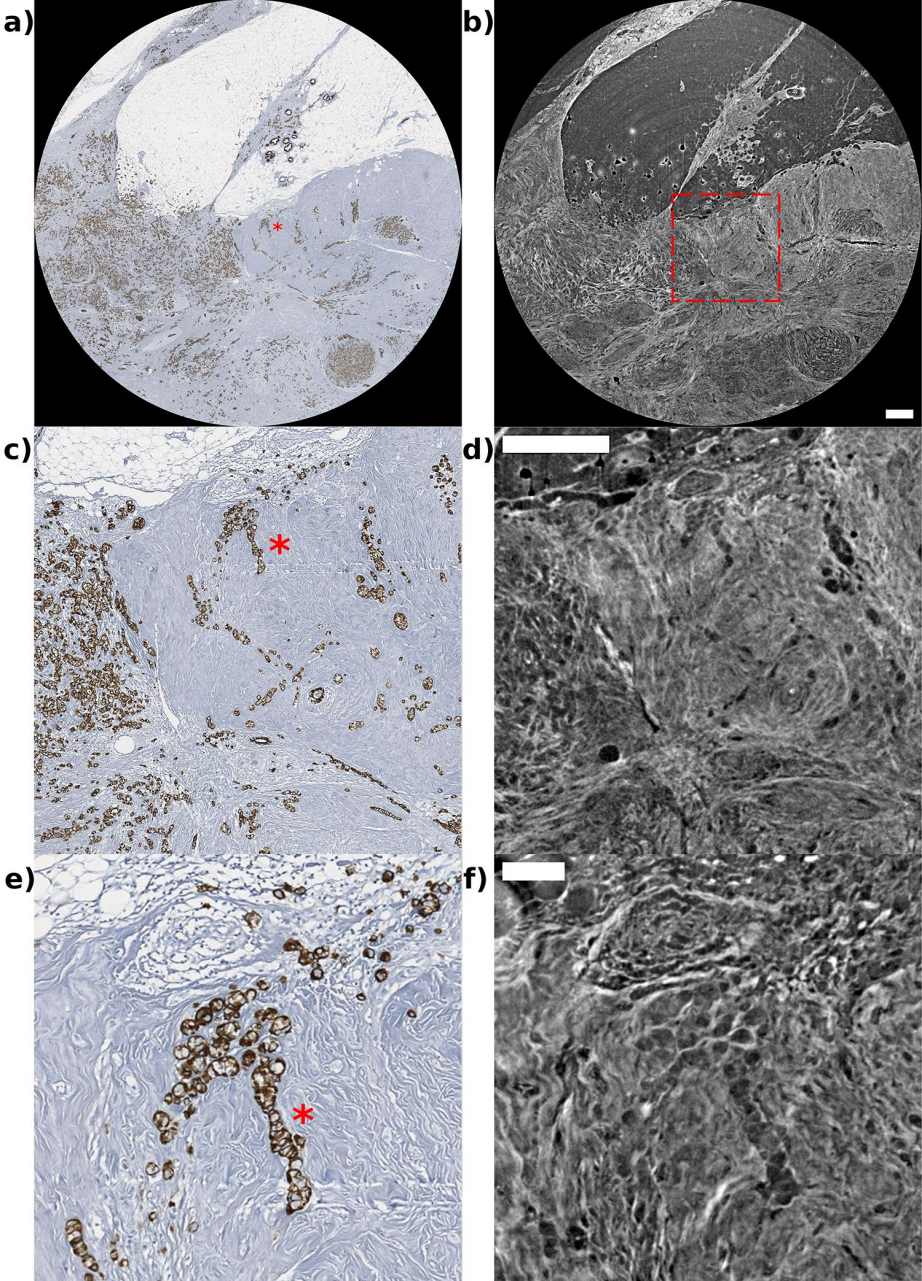
A comparison of the visibility of the features of tumor cells of ILC. On the left side are images with pancytokeratin histological coloring and on the right side are images of PhC taken at 4 μm (**b**,**d**) and 1 μm (**d**) respectively. (**b**) Shows the full field-of-view $\mu$CT slice, while panel d is the enlargement of region inside the red dashed contour line. 1 μm $\mu$CT images (f) are able to perfectly identify the spaces occupied by tumor cells in the stroma. Scalebars corresponding, respectively, to 0.5 mm for (**b**) and (**d**), and 0.1 mm for (**f**). On the pancytokeratin images are visible tumor cells arranged in single files, cords, and single cells (red asterisk) surrounded by abundant dense extracellular matrix. In the 1 μm image (e) it can be seen the single collagen fibers surrounding the neoplastic epithelial elements.

The tendency of the ILC to have atypical imaging is related to its histopathologic features and its inability to elicit a desmoplastic response as in ductal carcinoma. In ILC, the characteristic pattern of growth involves the infiltration of single cells or single files of cells through the stroma, with little disturbance of normal tissue architecture, with a tendency to spread between the collagen fibers of the breast and produce little desmoplastic response. ILC sample was scanned at 4 μm and 1 μm. Tomographic acquisitions were compared with images obtained with histological stainings (H&E in Fig. 4a, and pancytokeratin in Fig. 5a,c,e. The characteristic single-filed tumor cells of this type of breast lesion, known as Indian files, are indicated by the red asterisks in Fig. 5a,c,e. It is clearly visible in Fig. 5 that the tumor cells do not form solid, uniform lesion, but the cells are rather arranged between collagen fibers, that are prominently evident in the $\mu$CT images. Concerning spatial resolution, while in some cases the smallest pixel size (1 μm) might not be needed to identify key pathological structures, as for the micropapillary cystic carcinoma sample, for other lesions it is required to delineate small structures, as in the case of the filed tumor cells in the ILC presented in Fig. 5.

## Discussion

This study introduces a virtual histological approach using PhC $\mu$CT scans of human breast tissue samples, focusing on the visibility of pathologically relevant details. It shows its compatibility with the conventional histology workflow while offering advantages such as the 3D representation of the tissue specimen. The 3D data obtained through PhC $\mu$CT enables precise determination of the cutting position in a biopsy cassette, where finding a critical feature, such as infiltration centers in extensive in situ carcinomas, may alter the staging of the neoplasm. This approach has the potential to eliminate the need for multiple serial sections stained with H&E and immunohistochemical tests to assess basal membranes, thereby reducing the technical workload and the microscope viewing of numerous slides. A convincing spatial correlation was observed in all samples under examination (see, for example, Figs. 1, 2, 3, 4, and 5). Upon a detailed comparison, PhC $\mu$CT images exhibited a tissue morphology matching histology, resulting in nearly perfect overlapping details. Very minor morphological variations can be attributed to imperfect alignment of the 3D data to the histological image in the post-processing or deformations during sectioning and staining. Specifically, during the sectioning process, there is a possibility of losing small tissue fragments within hollow structures, such as mammary ducts or cystic formations. For instance, from Fig. 3, it can be appreciated that PhC $\mu$CT technique reveals accumulations of proteinaceous material and erythrocytes in microcystic structures (blue stars), which are not visible in the histological sections due to loss during the staining process. However, when comparing samples with low air cell presence and abundant extracellular matrix (see Figs. 4 and 5), the results are nearly identical. Moreover, PhC $\mu$CT proves its capability to identify individual collagen bundles surrounding neoplastic elements, as demonstrated in the panels c and d of Fig. 5. Already at lower magnification (Fig. 5), PhC $\mu$CT provides better visualization of the extracellular matrix compared to H&E staining and it is comparable to Masson’s trichrome staining (Fig. 1). Notably, in Fig. 1, PhC $\mu$CT accurately highlights the interlobular matrix and emphasizes the higher fiber density near the TLDU, which has gained recent recognition for its significance because the tissue microenvironment plays a crucial role in cancer formation, progression, and metastasis^40^. Furthermore, it is noteworthy that individual adipocytes are easily delineated, whereas in the histological section, the cellular membranes have been disrupted during the cutting and staining process.

Combining histological and radiological images has long been an integral part of the clinical diagnostic and therapeutic process. Nonetheless, each approach has its own limitations, which are also evident in our samples. PhC $\mu$CT is unable to discern and describe cell nuclei, a crucial aspect in diagnosing neoplastic conditions. To address this limitation, nano CT can be utilized, which allows for the observation of details at resolutions of a few hundreds of nanometers. However, using nano CT comes with the trade-off of a restricted field of view (< 1 mm) or a major increase in the number of scans required to cover large volumes, making it unpractical for clinical applications. On the other hand, conventional histology gives information about a single (or few) section(s) of the sample under examination. Even when histological serial sectioning is used, the spatial resolution achieved along the depth of the sample is much worse with respect to the in slice resolution. Moreover, being a destructive procedure, serial sectioning prevents the possibility of further analysis along different cutting planes.

**Figure 6:**
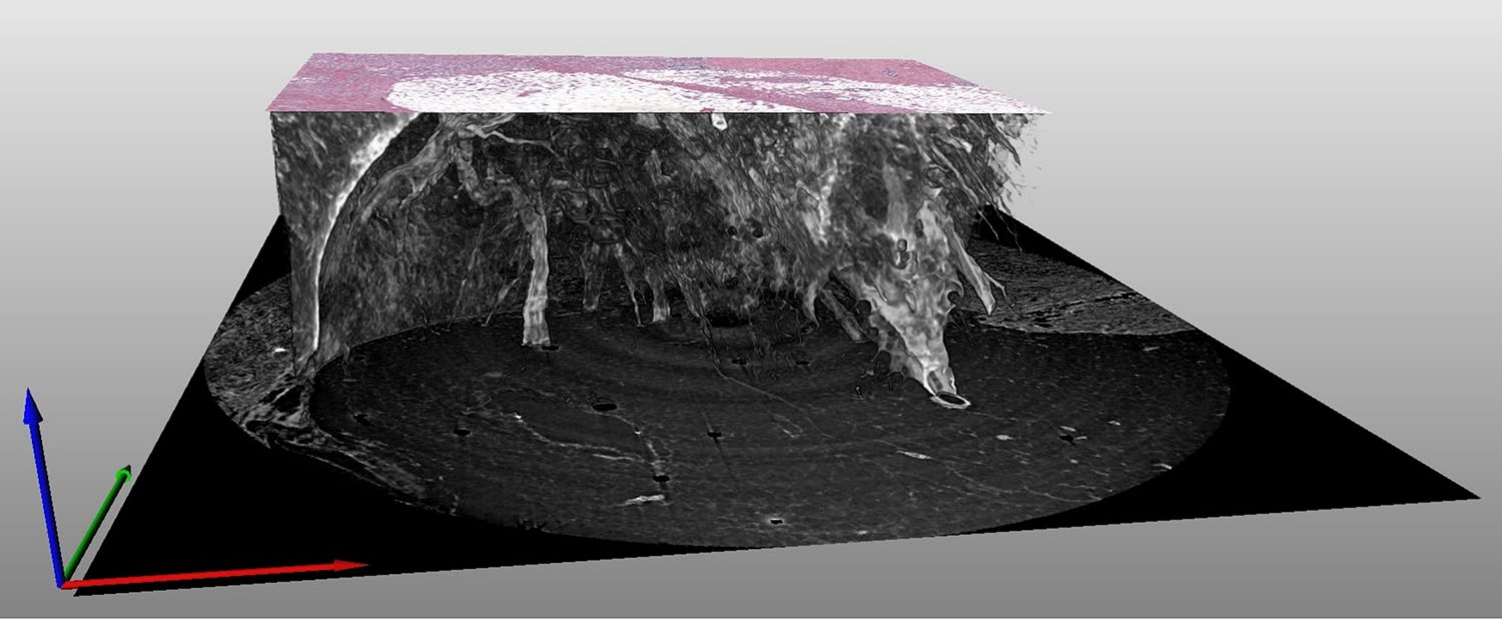
Hybrid visualization of conventional 2D histology slide and 3D PhC virtual histology image data volume of the invasive lobular carcinoma specimen. The area of the bottom slice is equal to $\sim$ 8 mm $\times \sim$ 8 mm, while the histological cut slice area is 2.4 mm $\times$ 1.4 mm (the thickness is around 5 μm). The rendered portion of the specimen volume is: 2.4 mm $\times$ 1.4 mm $\times$ 1.2 mm. Comparing the size of the images obtained with the two techniques it becomes evident that the histological section is representative of only a very small portion of the sample analyzed. The histological section is co-registered with the corresponding PhC $\mu$CT slice showing that images obtained with the two techniques can be combined and/or fused together. The volume rendering has been generated by using Avizo^®^ 3D software version.

In this context, the 3D potential of PhC $\mu$CT is shown in the volume rendering of the tissue included in the paraffin block in Fig. 6. The figure shows how the pathologist can visualize only a small portion of the tissue (H&E slice on top of the rendered volume): the prepared histological section is representative approximately of one-thousandth of the thickness of the tissue. According to the type of tissue to be studied, the structures in the cut planes below the first section, could be different from the ones analyzed in the selected plane section. There could be relevant features and complementary information that can support the pathologists in making the diagnosis that could get lost. In this framework, the use of PhC virtual histology could aid pathologists in examining the complete paraffin block and selecting the best cross-section of the pathological area of interest. This could be particularly beneficial in evaluating the invasiveness of carcinomas.

Although the proposed investigation is a proof of concept on a limited number of samples, the results reveal a promingsly high image quality and they facilitated the precise identification of morphological components and detailed characterization of tissues. Such capabilities hold significant potential for a broad spectrum of breast-related pathologies. It should be considered, however, that synchrotron facilities are intended for research purposes and are not widely available for clinicians. The case of the SYRMEP beamline at the Elettra synchrotron in Trieste (Italy), demonstrates the feasibility of using synchrotron radiation for 3D histology in clinical settings, especially when synchrotrons are located nearby, but it is a rare case. PhC imaging can be brought into clinical environments through two approaches. The first involves creating compact “synchrotron-like” X-ray sources capable of high spatial coherence and high flux^41^. The second approach entails utilizing different phase-contrast techniques that are compatible with conventional X-ray tubes^26,27,42–44^, despite their implementation typically entails longer scan times and/or worse spatial resolution. These translational studies reveal highly promising results, indicating their potential for future implementation in clinical practice.

## Conclusions

Through this research, the viability of the virtual histological approach using PhC $\mu$CT with breast tissue has been successfully demonstrated. The remarkable correspondence between conventional histology and PhC $\mu$CT images suggests that the latter could complement the histological characterization of these types of breast lesions. Conventional histology of paraffin-embedded tissue samples has been the gold standard for tissue analysis for decades. Although the multiple staining protocols and the microscopic resolution allow highly specific analysis down to the (sub)cellular level, histology falls short when it comes to obtaining 3D information about tissues. Serial sectioning marginally circumvents this problem, but is very time-consuming, labor intensive, and does not provide true 3D data. Conventional histology could benefit from the use of 3D volumes obtained with PhC $\mu$CT since the bi-dimensionality of the histological slides may limit the understanding and relationship of the original three-dimensional sample structure, thus potentially missing clinically relevant features. In this context, 3D virtual histology could support the missing gap in histological procedures by providing a global representation of the tissue, thus potentially guiding the pathologist to choose the most suitable direction prior to section cutting to prevent information loss, as well as providing additional morphological information during the evaluation of surgical margins towards breast-conserving surgery. PhC $\mu$CT technique also improves some image characteristics, resulting, for instance, in an enhanced visibility of the TDLU in the normal breast tissue compared to any of the three histological coloring methods. A greater definition of the complex arborizing fibrovascular cores, the widely accepted histological features of breast papillomas, was observed in PhC $\mu$CT images in contrast to H&E images, where additional staining was required for their perception. Moreover, the stromal/epithelial interface and the basement membrane, the latter being a key indicator of breast cancer progression, was highly contrasted in the virtual histology images of the micropapillary cystic carcinoma. Although PhC $\mu$CT lacks sufficient spatial resolution to allow a pathologist to distinguish infiltrating malignant tissues at a cellular level, as it is possible in conventional histology, it is still possible to observe encapsulated structures and elongated space occupied by the tumor cells. By highlighting the potential clinical role of 3D X-ray virtual histology, this study contributes to the growing body of knowledge in bio-imaging, demonstrating the added value of PhC $\mu$CT in the study of breast tissue alterations, and showcases the practical applications of micrometer-scale resolution in providing valuable morphological information for breast tissue analysis and disease diagnosis. This novel method will be used for future work on assessing the invasiveness of malignant diseases not only for breast but also in other cancers, such as the follicular thyroid carcinomas. This technique also has the potential to prompt future interdisciplinary collaborations focused on evaluating chronic pathologies, particularly fibrotic conditions, such as pulmonary fibrosis. However, it is the authors’ belief that virtual histology is not intended to replace traditional histopathology entirely. While digital slides can provide valuable information, certain diagnostic procedures, such as immunohistochemistry and molecular testing, may still require physical tissue sections. It is worth noting that while X-ray PhC $\mu$CT is a powerful imaging technique, it requires specialized equipment and expertise to perform. The technology is still evolving, and ongoing research is focused on further improving its resolution, sensitivity, and applicability in various fields of study. Overall, virtual histology holds great promise in improving efficiency, accessibility, and collaboration in the field of pathology and has the potential to enhance diagnostic accuracy and patient care.

## Methods

**Figure 7:**
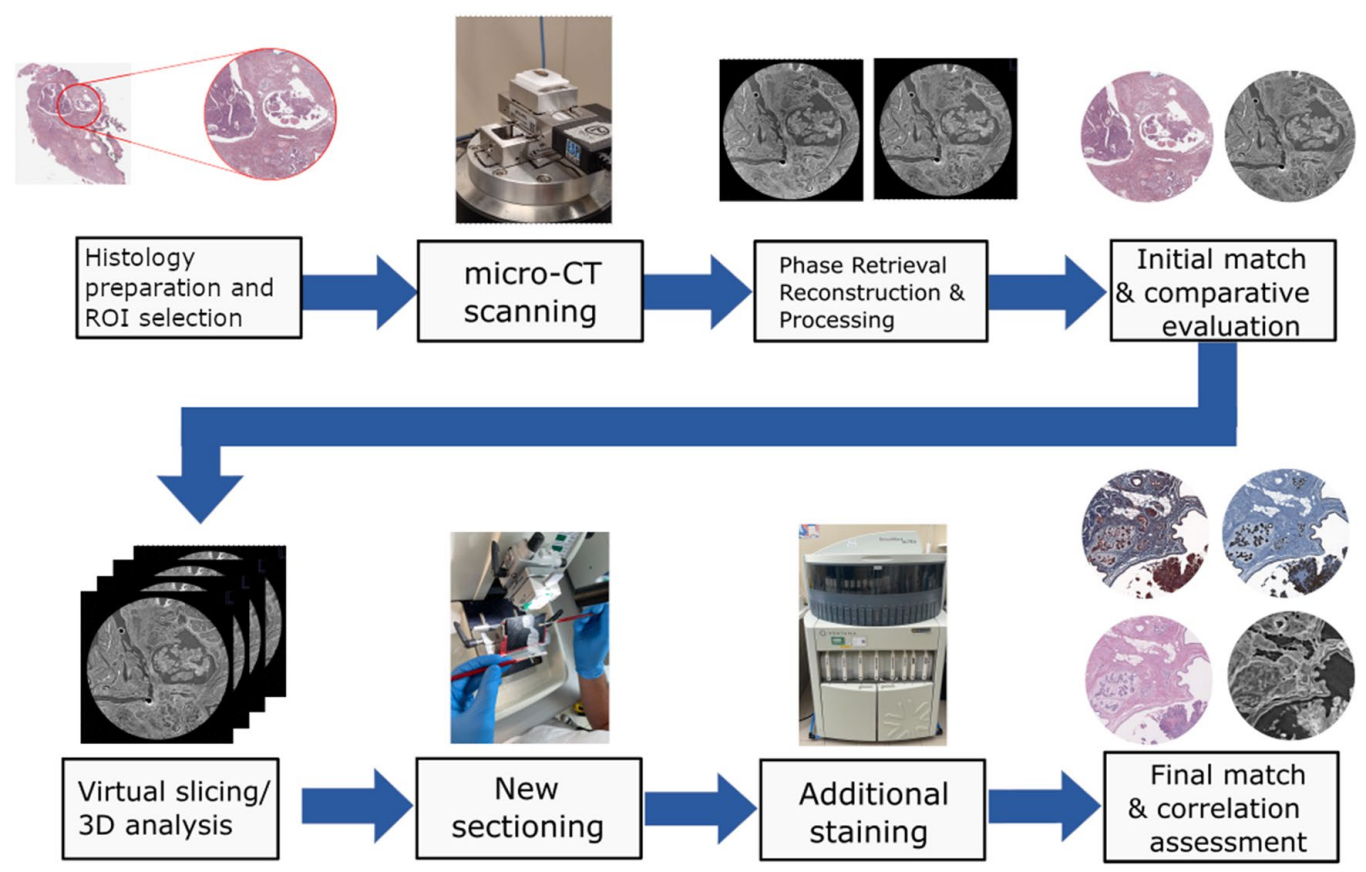
Step-by-step summary of the workflow followed in this study. After the characterization of the samples, selected regions of interest were scanned using X-ray PhC $\mu$CT. Next, the resulting projections were phase-retrieved, reconstructed, and analyzed. An initial match and a comparative evaluation have been made. New sections for additional histological staining procedures were posteriorly carried out. The resulting histological images were matched to their corresponding PhC $\mu$CT images. Finally, a compare-and-contrast assessment of the resulting matched images was done.

The procedure followed in the present study is schematically depicted in Fig. 7 and it will be detailed step-by-step in the following sections.

### Breast sample selection and preparation

**Figure 8:**
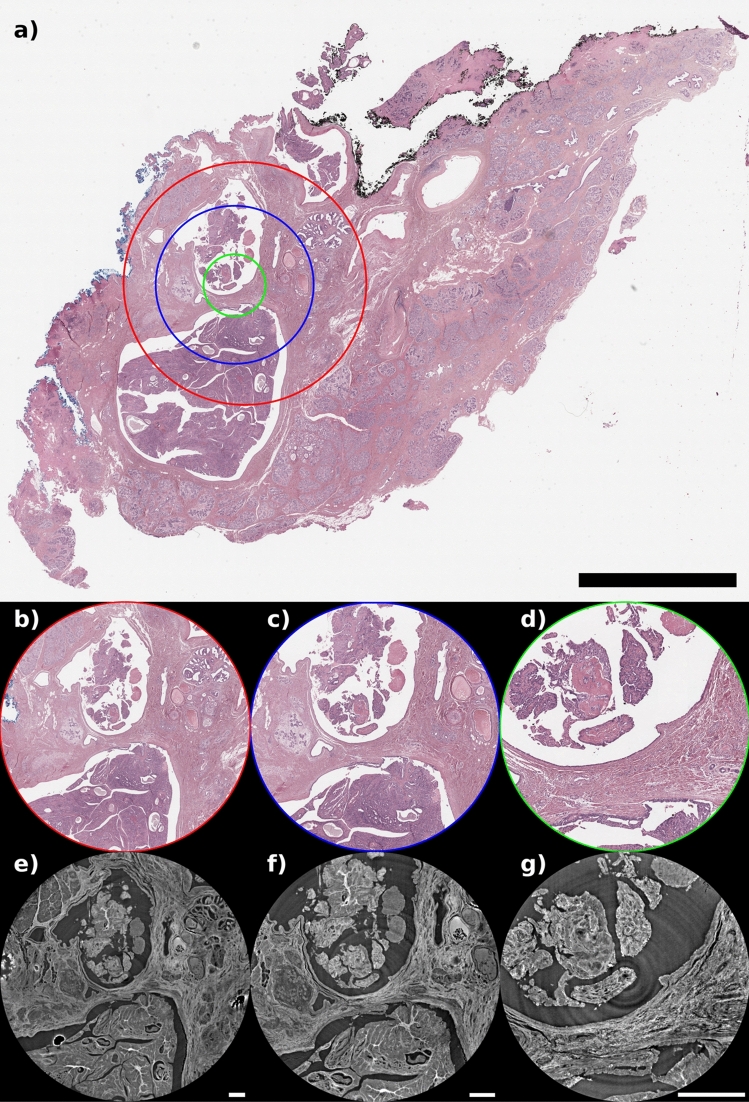
(**a**) H&E histological section of the intraductal papilloma tissue specimen. Scalebar equal to 5.0 mm. Red, blue, and green circles denote the field of view of the corresponding CT acquisitions at three different pixel sizes, respectively, 4.0, 2.5, and 1.0 μm. On the bottom panels the comparison of the same tissue portion as observed from histology and CT reconstructions at 4.0 μm (**b**,**e**), 2.5 μm (**c**,**f**) and 1.0 μm (**d**,**g**). Scalebars are equal to 0.5 mm.

Three breast tissue samples are selected from two lumpectomies and one mastectomy, following the framework of the operative protocol of the Breast Unit of the Trieste University Hospital (“PDTA Neoplasia mammaria”, approved on 11 December 2019 by ASUGI-Azienda Sanitaria Universitaria Giuliano Isontina, Italy) and the standard operative procedures of the clinical unit of the Anatomy and Histology Department of the University Hospital of Cattinara. The protocol entails written informed consent which is obtained from the patients before their inclusion in breast CT imaging studies. The specialist breast center of ASUGI is in compliance with the standard of EUSOMA guidelines (certificate No. 1027/01). The Directive 2004/23/EC of the European Parliament and of the Council of 31 March 2004 was followed on setting quality and safety standards for the donation, procurement, testing, processing, preservation, storage, and distribution of human tissues. All breast tissue samples undergo routine tissue processing for histology, fixed in 10$\%$ neutral-buffered formalin for 24 h. Subsequently, the fixed samples are processed (dehydrated in alcohol from 70 to 10$\%$, and cleared with xylene), and embedded in paraffin wax to create a tissue block (having a size of $3.0\,\text { cm} \times 2.5\,\text { cm }\times \sim 0.5\,\text { cm}$). The tissue blocks are sectioned with a microtome into approximately 4–5 micron sections and then placed on glass slides. The paraffin is removed from the tissue by a graded series of solvents, the tissue is then rehydrated and finally stained with H&E. Various stainings are useful for the study of mammary glands. The H& E combination is useful for the visualization of several structures: the hematoxylin component stains the nuclei in blue/purple, while the eosin gives a pinkish hue to eosinophilic structures (e.g., cytoplasm, collagen, and muscle fibers). Masson’s trichrome stains collagen fibers and is mostly used to evaluate fibrosis. Moreover, immunostaining can be used to identify antigens that serve as important diagnostic markers. All the histological images are digitized using a D-Sight F 2.0 slide scanner with the same acquisition conditions with a magnification of $20\times$ and pixel size of 0.5 μm × 0.5 μm. After the embedding, some volumes (selected from the inspection of the thin surface section by the pathologists) of histological interest in the tissue were chosen to be scanned using PhC $\mu$CT at different pixel sizes (see Fig. 8). The three acquired samples are representative of one benign breast lesion and two malignant lesions.

### Experimental $\mu$CT setup

X-ray PhC $\mu$CT acquisitions of the previously selected region of interests for each paraffin-embedded sample are performed at the SYRMEP beamline^34,45^ of the Italian synchrotron facility Elettra (Trieste, Italy), with a filtered (1.0 mm of Si) polychromatic beam having an average energy of 20 keV. Acquisitions were performed by using an sCMOS detector (Hamamatsu ORCA-Flash 4.0 model C11440-22C) featuring a 6.5 μm pixel size and a sensitive area of $2048\times 2048$ pixels. The sensor is coupled to a high numerical aperture magnifying optics allowing tuning the effective pixel size in the range from 1 to 6.5 μm. X-ray conversion is obtained through a 45 μm thick GGG:Eu scintillator. The imaging system at the beamline offers different scintillator options, allowing to balance resolution and sensitivity. Thicker scintillators enhance sensitivity but may sacrifice spatial resolution, whereas thinner ones provide sharper images at the cost of sensitivity. The aim of the experiment was to strike a compromise between rapid scan times and spatial resolution, ensuring efficient imaging without sacrificing detail. For this experiment, three effective pixel sizes of 1, 2.5, and 4 μm (corresponding to spatial resolutions of 3.0, 7.0 and 10.0 μm, respectively^28^), resulting in images of lateral field-of-view (FOV) of $\sim$ 2, $\sim$ 5, and $\sim$ 8 mm in diameter respectively, were employed (see Fig. 2). Considering the sensor size, acquisitions at 2.5 and 4 μm enabled the acquisition of the full height of the sample ($\sim$ 5 mm). When the pixel size is set to 1 μm a 2 mm high portion of the volume from the upper surface of the paraffin-embedded block was imaged. $\mu$CT scans were obtained by acquiring 1800 evenly spaced projections over a 180-degree angle in the propagation-based phase-contrast imaging modality^32,46^, leading to a total scan time of 72 seconds. This imaging modality requires a careful selection of the propagation (i.e. sample-to-detector) distance to maximize soft tissue visibility while retaining a high spatial resolution, which is dependent on the effective pixel size^46,47^. Following a previously validated optimization study^28^, the propagation distances were set to 500 mm for 4 μm pixel size, 250 mm for 2.5 μm pixel size, and 150 mm for 1 μm pixel size. The source-to-sample distance was fixed at 22.3 m. As the detector FOV is smaller than the lateral dimension of the samples, a local-area CT^48^ dataset centered on a region of interest selected by the pathologist was acquired for each specimen. A one-to-one comparison between histology and PhC $\mu$CT is shown in Fig. 8. Specifically, the upper panel shows the intraductal papilloma sample stained with H&E together with three circular regions of interest (ROIs) that represent the cross-sectional area acquired with PhC $\mu$CT at the three different pixel sizes. Red, blue, and green circles correspond to areas acquired at 4, 2.5, and 1 μm, respectively. The lower panels of Fig. 8 show the comparison of the histological image with the corresponding PhC $\mu$CT at each employed pixel size. It is worth mentioning that PhC $\mu$CT scanning does not require any specific sample preparation, it is fully compatible with further histological investigation, and, with the selected acquisition parameters, no noticeable radiation damage is observed^19,23^.

### $\mu$CT reconstruction and image processing

Acquired projections were pre-processed by conventional flat-fielding image correction and ring artifacts removal^49^. After preprocessing, the projections were phase-retrieved using the Homogeneous Transport-of-Intensity-Equation (TIE-Hom) algorithm^50^ and setting the filter parameter $\delta /\beta$ = 350, taking into account the interfaces of glandular and fat materials^51,52^ at the average energy of the spectrum and using the web service available at http://ts-imaging.science.unimelb.edu.au//Services//Simple//ICUtilXdata.aspx. Reconstruction was performed with a GPU-based filtered back projection (FBP) algorithm and Shepp-Logan filtering^53^. $\mu$CT reconstructions were post-processed by a de-trending procedure^54^ to mitigate cupping effects due to beam-hardening and phase-retrieval. After image processing, the final tomographic reconstructions provided a 3D visualization of the acquired region of interest along the depth of the whole sample. After processing, the final $\mu$CT reconstruction yields a 3D map which is proportional to the linear attenuation coefficient of the sample^55^. $\mu$CT slices shown in Figs. 2, 3, 4, 5 have been processed with Avizo® 3D using an unsharp masking filter^56^ to enhance fine tissue details.

### Virtual slicing, 3D assessment, and correlation with histology

Tomographic reconstruction slices corresponding to the surface of the paraffin-embedded sample block were reviewed and compared to the preliminary histological sections immediately after the tomographic reconstruction. PhC $\mu$CT reconstructions allowed an interactive virtual visualization of the 3D sample volume. The experienced pathologist reviewed the complete sample through virtual slicing and assessed the key features in the three orthogonal planes of the specimen. Volume analysis improves the pathologist’s understanding of specimen, allowing them to be guided for consecutive sectioning. New histological sections are then produced and, depending on the features that need to be emphasized, a specific staining technique was used (see the sample preparation section). Using the new histological images as a reference, the pathologist manually traced and matched the key features in the histology slide that best matched in the corresponding virtual $\mu$CT stack. Finally, CT reconstructions were broadly analyzed by the pathologist by comparing, contrasting, and correlating the visible morphological structures across the methodologies, as well as pin-pointing visualization advantages and disadvantages of PhC $\mu$CT images to the different staining protocols.

## Data Availability

All data generated or analysed during this study are included in this published article.
